# Child passenger safety education in the emergency department: teen driving, car seats, booster seats, and more

**DOI:** 10.1186/s40621-020-00250-5

**Published:** 2020-06-12

**Authors:** Cassi Smola, Annalise Sorrentino, Nipam Shah, Michele Nichols, Kathy Monroe

**Affiliations:** 1 Department of Pediatrics, Hospital Medicine Division, University of Alabama Birmingham, 1600 7th Ave So, Suite 110 CPP, Children’s of Alabama, Birmingham, Al 35233 USA; 2 Department of Pediatrics, Division of Pediatric Emergency Medicine, University of Alabama Birmingham, 1600 7th Ave So, Suite 110 CPP, Children’s of Alabama, Birmingham, Al 35233 USA

**Keywords:** Child passenger safety, Car seats, Booster seats, Teen drivers

## Abstract

**Background:**

The leading cause of death in children less than 19 years old is motor vehicle crashes (MVC). Non-use or improper use of motor vehicle car seats significantly adds to the morbidity and mortality. Emergency department (ED) encounters provide an opportunity for caregiver education. Our objective was to determine the effect of an educational intervention on knowledge and counseling behaviors of pediatric ED nurses regarding child passenger safety (CPS).

**Methods:**

A pre/post educational intervention study was conducted with nursing staff in an urban ED. Responses to CPS related knowledge and counseling behaviors were collected using surveys administered before and after the intervention. The ED nurse education intervention was a one-hour lecture based on the American Academy of Pediatrics (AAP) CPS guidelines and Alabama state law regarding ages for each car seat type and teen driving risky behaviors. Individual data from pre and post surveys were matched, and nominal variables in pre-post matched pairs were analyzed using McNemar’s test. To compare categorical variables within pre or post test data, we used the Chi-square test.

**Results:**

Pretests were administered to 83/110 ED nurses; 64 nurses received the educational intervention and posttest. On the pretests, nurses reported “never” or “occasionally” counseling about CPS for the following: 56% car seats, 62% booster seat, 56% teen driving, 32% seat belts. When comparing the pretest CPS knowledge between nurses working 0-1 year vs. ≥ 2 years there was no statistically significant difference. Two CPS knowledge questions did not show significance due to a high correct baseline knowledge rate (> 98%), including baseline knowledge of MVC being the leading cause of death. Of the remaining 7 knowledge questions, 5 questions showed statistically significant improvement in knowledge: age when children can sit in front seat, state GDL law details, seat belt state law for back seat riders, age for booster seat, and rear facing car seat age. All four counseling behavior questions showed increases in intent to counsel families; however, only intent to counsel regarding teen driving reached statistical significance.

**Conclusions:**

Educational efforts improved pediatric ED nursing knowledge regarding CPS. Intent to counsel was also improved following the education.

## Background

Eight million nonfatally injured patients seek care in emergency departments (EDs) annually in the United States (Durbin and Hoffman 2018a). While physicians overwhelmingly support counseling patients on topics surrounding injury prevention, significant barriers exist to its implementation with lack of training and limited confidence included as leading causes (Kwong et al. 2019). The Emergency Nursing Association position statement states ED nurses are poised to lead in the prevention of injury through evidence-based education, public education, and healthcare advocacy (ENA position statement n.d.). The National Highway Traffic Safety Administration (NHTSA) has called for hospital-based programs for child passenger safety (CPS) education in hospital discharge planning to include all health care team members (NHTSA n.d.). Despite these recommendations, prior studies indicate injury prevention counseling by emergency staff is varied and implemented inconsistently (Wilding et al. 2008).

Roger et al. conducted a pre-post intervention study to evaluate an educational child safety seat (CSS) program for nurses in maternal/newborn unit in the post-partum period (Rogers et al. 2013). To our knowledge, there are no studies about knowledge of nurses regarding CPS practice for children of all age groups and if an educational program will improve nurse education. The objective of this study was to determine the baseline knowledge of ED nurses, if experience (years of nursing) had an impact on counselling behaviors, and to determine the impact of a targeted CPS education upon both knowledge and intention to counsel patients and families on CPS. Our hypothesis was that a brief educational intervention would lead to change in nurses’ knowledge and intent to counsel about CPS.

## Methods

### Study design and setting

A pre/post intervention study was conducted at Children’s of Alabama (COA) pediatric emergency department in February 2018. Our institution is a tertiary care children’s hospital with over 73,000 patients seen annually in our emergency department. Of these, 400 cases are trauma related, and of those, about 50% are due to MVC. The subjects served as their own controls with the pre-test being conducted prior to any educational effort. The post test was conducted following the CPS educational intervention to determine effectiveness of the education. The study was deemed exempt (as an educational intervention) by Institutional review board of University of Alabama at Birmingham.

### Survey development and content

**Fig. 1:**
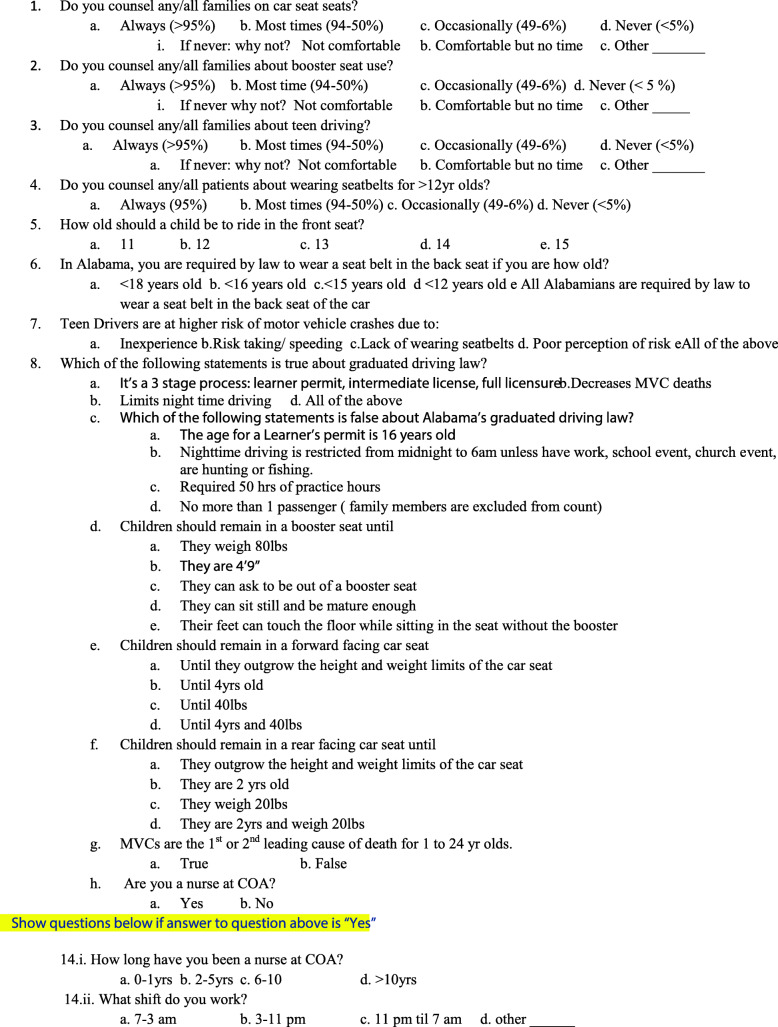
Child Passenger Safety Pre- and Post-Test

Pre and post intervention tests (same questions) were initially drafted by pediatric faculty. The test was developed by the principal investigators, who are experts in injury prevention, and specifically in child passenger safety. Three of the investigators have been practicing pediatric emergency medicine physicians for over 20 years each. These three are the leaders of the resident education rotation in injury prevention at our institution and have extensive experience teaching the residents the concepts of CPS. Test questions were based on the American Academy of Pediatrics (AAP) guidelines on child passenger safety (Durbin and Hoffman 2018b; Committee on Injury Violence and Poison Prevention 2011). Questions were included regarding: 1) appropriate ages for car seats (rear and forward facing), booster seats, and front seat riding; 2) risky teen driving behaviors; and 3) elements of the state graduated driver licensing (GDL) laws (Child Restraints n.d.) (Fig. 1). The initial survey drafts were reviewed and edited by four pediatric faculty and after discussion and consensus, a revised draft was developed. Both surveys consisted of 15 questions including: 9 knowledge questions regarding CPS, 2 questions about how long the participants had been nurses and which shifts they typically worked, and 4 questions asking how often nurses counseled families regarding car seats, booster seats, seat belts, or teen driving. The counseling behavior response options for these questions were on a Likert scale as: Always, Most times, Occasionally and Never. There were follow up questions for all the counseling questions for nurses to answer to help us further understand why they did not counsel. The response options to follow up questions included: not comfortable, no time, and other.

### Survey administration

Flyers were distributed in the pediatric ED before initiating the study. Nursing huddles are conducted daily in the department, and these huddles were used to provide nurses information regarding the study. Verbal consent was obtained from the nurses to participate in this study. During these huddles, the pre-intervention test was provided to nurses in paper form, and an email with a link to an online version was sent to all nurses as well. Nurses were given 1 week to complete the survey. This was followed by the educational intervention during a department wide educational event for 2 weeks. The post-intervention survey was given to nurses in paper format at the end of the educational intervention session.

### Educational intervention

**Fig. 2:**
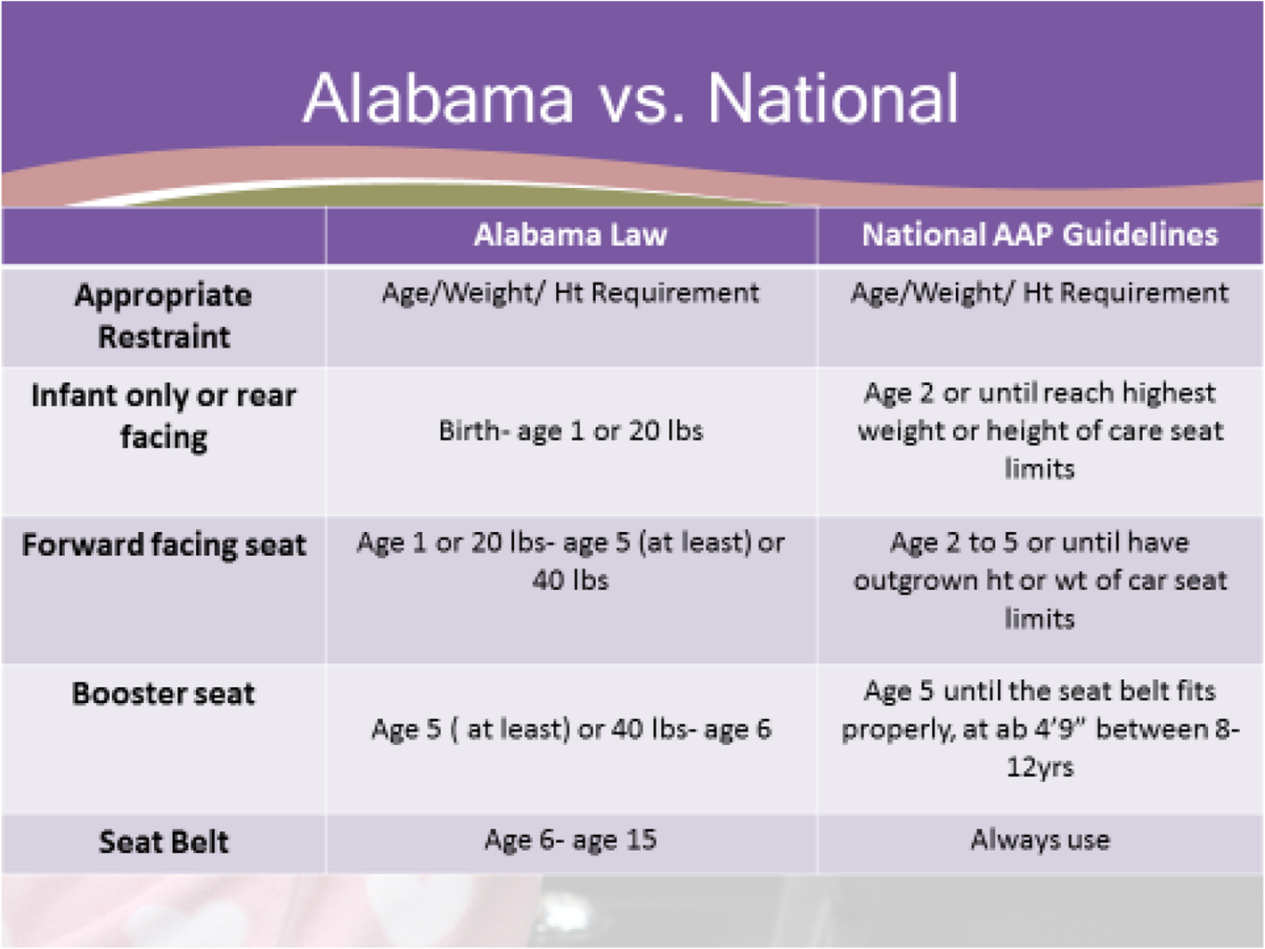
Differences in Alabama Law vs Best Practice AAP Guidelines

The educational intervention was provided in a one-hour lecture format with a slide presentation. The sessions were part of the hospital’s biannual nursing education events, which were held on four separate days to allow nursing staff an opportunity to attend. The intervention was presented on all 4 days. The sessions were moderated by the study authors, who presented the following topics: 1) rear and forward facing car seat age guidelines 2) booster seat age guidelines, 3) seat belt age guidelines, and 4) front seat passenger age requirements as listed in the AAP policy and technical report on child passenger safety. The specific state law was also addressed, which differs from the AAP guidelines in the ages from which children can graduate from each type of car seat (Fig. 2). The sessions ended with an interactive a question and answer period at the end of the intervention.

### Statistical methods

For purposes of analysis, responses were re-categorized in some of the questions. For instance, the Likert scale response categories in first 4 behavior questions were re-categorized into binary response (i.e. Always/Most of the times and Occasionally/Never). Similarly, we categorized nurses as younger nurses with ≤ one-year experience and older nurses with more than ≥ 2 years nursing experience. We calculated descriptive statistics, including frequencies and proportions. Data from pre and post-tests were matched using unique codes for each participant, and nominal variables in pre-post matched pairs were analyzed using McNemar’s test. To compare categorical variables within pre or post test data, we used the Chi-square test. Significance level was set at 0.05. All analyses were conducted using SPSS version 24.0 (IBM Corp, Armonk, NY).

## Results

**Fig. 3:**
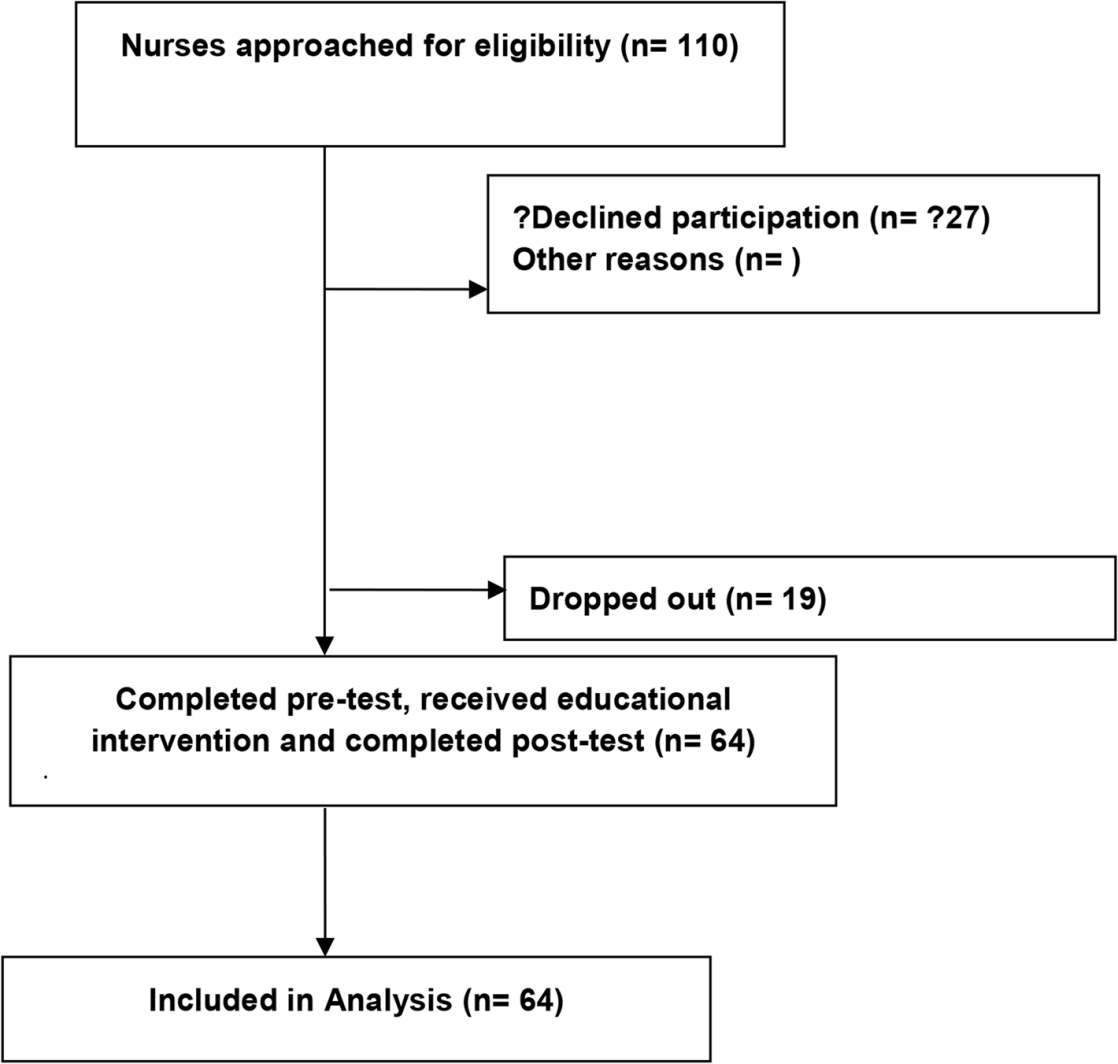
Study Flow Diagram

A total of 83 out of 110 ED nurses (75%) completed the pretest survey. Of those, 64 participants (77%) received the educational intervention and completed the post tests. Nineteen (22%) of the original nurses were excluded secondary to incomplete data collection (Fig. 3). Of the original 83 participants, 34% had been a nurse for 0–1 year and of the 64 participants completing the survey, 41% had been a nurse for 0–1 year.

The 64 participants who completed both the pre- and post -tests were queried as to their behaviors related to motor vehicle safety counselling for their patients. In many of the pretests, nurses reported “never” or “occasionally” counseling about CPS (69% car seats, 78% booster seat, 88% teen driving, 42% seat belts) (Table 1). Data regarding counseling by experience were analyzed as noted in Table 2. Only for counselling regarding booster seats was there a statistically significant difference between the more experienced and less experienced nurses. Reasons for not counseling included not being comfortable doing so or not having enough time. After the intervention, all questions regarding the intent to counsel families demonstrated an increased intent by the nurses, but only the teen driving intent to counsel reached statistical significance (*p* = 0.01).

**Table 1:** Pre-test and Post-test: Counseling Frequency of Families on CPS by Type of Education

Pre-test	Always	Most times	Occasionally	Never	Always or most times	Occasionally or Never
**Car Seat**	7 (10.9%)	13 (20.3%)	29 (45.3%)	15 (23.4%)	20 (31.3%)	44 (68.8%)
**Booster Seat**	6 (9.4%)	8 (12.5%)	31 (48.4%)	19 (29.7%)	14 (21.9%)	50 (78.1%)
**Teen Driving**	2 (3.1%)	6 (9.4%)	28 (43.8%)	28 (43.8%)	8 (12.5%)	56 (87.5%)
**Seat Belts**	16 (25%)	21 (32.8%)	20 (31.3%)	7 (10.9%)	37 (57.8%)	27 (42.2%)
**Post-test**	**Always**	**Most times**	**Occasionally**	**Never**	**Always or most times**	**Occasionally or Never**
**Car Seat**	7 (10.9%)	19 (29.7%)	28 (43.8%)	10 (15.6%)	26 (40.6%)	38 (59.4%)
**Booster Seat**	6 (9.4%)	14 (21.9%)	29 (45.3%)	15 (23.4%)	20 (31.3%)	44 (68.7%)
Teen Driving*	3 (4.7%)	19 (29.7%)	25 (39.1%)	16 (25%)	22 (34.9%)	41 (64.1%)
**Seat Belts**	12 (19%)	24 (37.5%)	22 (34.4%)	5 (7.8%)	36 (57.1%)	27 (42.2%)

**Table 2:** Pre-test: Counseling Frequency by Nursing Experience

Counseling	< 1 year	≥ 2 years	*p* value
**Car Seats**	6/ 26 (23.1%)	14/38 (36.8%)	0.24
**Booster Seats**	3/26 (11.5%)	11/38 (28.9%)	0.09
**Teen Driving**	3/26 (11.5%)	5/38 (13.2%)	0.85
**Seat Belts**	13/26 (50%)	24/38 (63.2%)	0.29

In the pre-test (and post-test), the two knowledge questions, which most nurses answered correctly (> 98%) were about reasons teen drivers were at higher risk of being involved in an MVC and knowledge of MVCs being the leading cause of death in children 0–24 years old (Table 3). The other seven questions addressed state motor vehicle passenger seat belt laws, high risk teen drivers, and components of graduated driver’s licensing, booster seat age requirements, and forward and rear facing car seats age guidelines. Of these seven knowledge questions, five questions showed statistically significant improvement after the intervention in knowledge (Table 3).

**Table 3:** Comparison of behavior and knowledge Pre and Post intervention

Variable	Pre-test	Post-test	Percentage difference^a^	*p* value
**Counseling of patients**				
Car seat	20/64 (31.3%)	26/64 (40.6%)	+ 9.3%	0.31
Booster seat	14/64 (21.9%)	20/64 (31.3%)	+ 9.4%	0.26
Teen driving	8/64 (12.5%)	22/64 (34.4%)	+ 21.9%	**0.01**
Seat belts	37/64 (57.8%)	36/64 (56.3%)	+ 1.5%	1.00
**Knowledge**				
Front seat rider	20/64 (31.3%)	53/64 (82.8%)	+ 51.5%	**< 0.0001**
Back seat belt	38/64 (59.4%)	11/64 (17.2%)	−42.2%	**< 0.0001**
High risk teen drivers	63/64 (98.4%)	63/64 (98.4%)	0.0%	1.00
GDL components	55/64 (85.9%)	61/64 (95.3%)	+ 9.4%	0.11
GDL law	39/64 (60.9%)	59/64 (92.2%)	+ 31.3%	**< 0.0001**
Booster seat	22/64 (34.4%)	37/64 (57.8%)	+ 23.4%	**0.02**
Forward facing	33/64 (51.6%)	40/64 (62.5%)	+ 10.9%	0.21
Rear facing	20/64 (31.3%)	34/64 (53.1%)	+ 21.8%	**0.01**
MVC leading cause of death	63/64 (98.4%)	64/64 (100%)	+ 1.6%	–

^a^(“+” denotes increase from pre to post and “- “denotes decrease from pre to post)

## Discussion

Our study found an educational intervention significantly increased nurses’ CPS knowledge and intent to counsel. Baseline knowledge was excellent for the questions regarding the motor vehicle crashes being the number one cause of injury and teen driving behavior risks. The remaining knowledge questions focused on state passenger seat belt, car seat and booster seat age legislation also showed significant improvement after the intervention. The intent to counsel questions found over 50 % of nurses reporting they had never or only occasionally counseled about car seats, booster seats or teen driving safety (69, 78 and 87% respectively). After the intervention, intent to counsel percentages increased for all CPS categories.

Our ED had had a high turnover of nursing staff in recent year, and 34% of the nursing staff participants had been practicing nurses for less than or equal to a year. We anticipated at baseline those newer nurses would be less knowledgeable about CPS guidelines and the law; however, we did not find any significant differences in knowledge. Our findings may be due to the prior outreach efforts in the state regarding MVC safety and teen driving safety.

In our sample of ED nurses, we found baseline injury prevention CPS counseling behaviors were low overall. While nurses in our study were more likely to counsel families on seat belt use (versus other areas of cps counseling), 42% reported doing this only occasionally or never. Our nurses listed time and comfort level as the main barriers to counseling. In terms of using the ED as a potential educational environment, Chun et al. found significant predictors for health care professionals to counsel regarding alcohol abuse while in the ED were previous counseling training and prior counseling experience (Chun et al. 2011). Similar studies found registered nurses were less likely to feel confident about their counseling abilities if they had not been adequately educated and trained on the topic (Johanson et al. 2005; Aalto et al. 2001). Lack of training and confidence in administering behavioral interventions were identified as barriers to effective counseling (Chun et al. 2011). Following our intervention, intent to counsel families increased in all four of the behavioral questions in both groups. Even though only counseling about teen driving reached statistical significance, we feel the increase overall to be a positive finding.

It is important to educate families on the appropriate use of CPS as use of appropriate CPS restraints has been shown to reduce injury in infants involved in MVCs (Agran et al. 1998; Mr et al. 2006; Huang et al. 2016). In 2011 the AAP began recommending that infants and children less than 2 years old be restrained in a rear facing seat in the vehicle’s rear seat. Despite AAP guidelines and the strengthening of child restraint laws, an unacceptably low proportion of infants and toddlers are being transported in accordance with current best practice (Huang et al. 2019). The AAP updated the policy statement on CPS again in 2018, with the current recommendations stating children should remain rear facing for as long as allowable by the car seat manufacturer guidelines (Durbin and Hoffman 2018b; Committee on Injury Violence and Poison Prevention 2011). CPS guidelines are detailed, and recent changes to best practice may have contributed to some confusion. Most state laws lag behind best practice recommendations; therefore, focusing on best practice guidelines should be the goal. Putting resources toward the use of a standard injury prevention screening tool has been shown to improve injury prevention in primary care offices. (Gittelman et al. 2018) It stands to reason that the ED may be another setting for this type of tool to be utilized.

### Limitations

This study was conducted in a free-standing pediatric emergency department in a single-center southern academic institution, which may limit our generalizability. Not all the ED nurses participated in and completed the study. Also, this was self-reported data collection, which may be subject to recall and social desirability bias. As all the reporting was anonymous and voluntary, we feel this was less likely to impact the findings. The intent to counsel families is different from nurses actually counseling families on CPS. This study only measured intent to counsel. Future studies observing counselling behavior should be considered. Finally, the post-test was administered immediately after the intervention; therefore, long term knowledge retention and counselling intent and behavior are unknown.

## Conclusions

The ED nurses in our sample demonstrated high rates of some baseline knowledge of motor vehicle crash risks, but a low report of CPS counselling to families in the ED. A brief educational intervention improved pediatric emergency nurses’ knowledge and intention to counsel families on child passenger safety topics. Future studies would be important to determine if this educational intervention changes long term counselling behavior.

## Data Availability

We are happy to make our data and surveys available upon request.

## Abbreviations

AAP: American Academy of Pediatrics
COA: Children’s of Alabama
CPS: Child passenger safety
CSS: Child safety seat
ED: Emergency department
GDL: Graduated driver licensing
MVC: Motor vehicle crash
NHTSA: National Highway Traffic Safety Administration

## References

[CR1] Aalto M, Pekuri P, Seppa K (2001). Primary health care nurses and physicians attitudes, knowledge and beliefs regarding brief intervention for heavy drinkers. Addiction..

[CR2] Agran P, Anderson C, Winn D (1998). Factors associated with restraint use of children in fatal crashes. Pediatrics.

[CR3] Child Restraints: Car Seats and Booster Seats found at the Alabama Public Health web site https://www.alabamapublichealth.gov/injuryprevention/car-seats.html. Accessed 19 Dec 2019.

[CR4] Chun T, Spirito A, Rakowski W, Onofrio G, Woolard R (2011). Beliefs and practices of pediatric emergency physicians and nurses regarding counseling alcohol using adolescents. Peds Emerg Care.

[CR5] Committee on Injury Violence and Poison Prevention (2011). AAP technical Report on Child Passenger Safety. Pediatrics.

[CR6] Durbin D, Hoffman B (2018). Child Passenger Safety. AAP policy statement from Council on Injury Violence and Poison Prevention. Pediatrics.

[CR7] Durbin DR, Hoffman BD (2018). Council on Injury, Violence, and Poison Prevention. Child Passenger Safety. Pediatrics.

[CR8] ENA position statement: The role of the emergency nurse in injury prevention. Available at https://www.ena.org/docs/default-source/resource-library/practice-resources/position-statements/injuryprevention.pdf?sfvrsn=8242c4a2_12. Accessed 28 July 2019.

[CR9] Gittelman MA, Carle AC, Denny S, Anzeljc S, Arnold MW (2018). A quality improvement program in pediatric practices to increase tailored injury prevention counseling and assess self-reported changes made by families. Injury Epidemiology.

[CR10] Huang Y, Liu C, Pressley JC (2016). Child restraint use and driver screening in fatal crashes involving drugs and alcohol. Pediatrics.

[CR11] Huang Y, Lui C, Pressley J (2019). Restraint use and injury in forward and rear-facing infants and toddlers involved in a fatal motor vehicle crash on a U.S. roadway. Inj Epi.

[CR12] Johanson K, Akerlind I, Bendtsen P (2005). Under what circumstances are nurses willing to engage in brief alcohol interventions? A qualitative study from primary care in Sweden. Addict Behav.

[CR13] Kwong JZ, Gray JM, Rein L, Liu Y, Melzer-Lange MD (2019). An educational intervention for medical students to improve self-efficacy in firearm injury prevention counseling. Injury Epidemiol.

[CR14] Mr E, Kallan MJ, Durbin DR, Winston FK (2006). Effectiveness of child safety seats vs seat belts in reducing risk for death in children in passenger vehicle crashes. Arch of Ped and Adol Med.

[CR15] NHTSA: Hospital discharge recommendations for safe transportation of children, emphasize that an effective hospital-based program should include physicians, nurses, therapists, administrators, risk management staff and community outreach team members. https://www.nhtsa.gov/sites/nhtsa.dot.gov/files/documents/812106_hospitaldischrgerecsafetranschildren.pdf. Accessed 10 Sept 2019.

[CR16] Rogers SC, Gallo K, Saleheen H, Lapidus G (2013). Can nurse education in the postpartum period reduce car seat misuse among newborns?. J Trauma Acute Care Surg.

[CR17] Wilding L, O'Brien JA, Pagliarello G, Friedberg E (2008). Survey of current injury prevention practices by registered nurses in the emergency department. J Emerg Nurs.

